# Integrated Metabolomics and Transcriptomics Unravel the Metabolic Pathway Variations for Different Sized Beech Mushrooms

**DOI:** 10.3390/ijms20236007

**Published:** 2019-11-28

**Authors:** Su Young Son, Yu Jin Park, Eun Sung Jung, Digar Singh, Young Wook Lee, Jeong-Gu Kim, Choong Hwan Lee

**Affiliations:** 1 Department of Bioscience and Biotechnology, Konkuk University, Seoul 05029, Korea; syson119@naver.com (S.Y.S.); y_zing@naver.com (Y.J.P.); singhdigar@gmail.com (D.S.); 2 Department of Systems Biotechnology, Konkuk University, Seoul 05029, Korea; jes708@naver.com; 3 Bumwoo Mushroom Farm Co. Ltd., 538 Yeoyang 1-ro, Yeoju 12609, Korea; bumwoo0789@naver.com; 4 Genomics Division, National Academy of Agricultural Science, Rural Development Administration, Jeonju 54874, Korea; 5 Research Institute for Bioactive-Metabolome Network, Konkuk University, Seoul 05029, Korea

**Keywords:** beech mushroom, different sizes, metabolomics, transcriptomics, metabolic pathway

## Abstract

Beech mushrooms (*Hypsizygus marmoreus*) are largely relished for their characteristic earthy flavor, chewy-texture, and gustatory and nutritional properties in East Asian societies. Intriguingly, the aforementioned properties of beech mushroom can be subsumed under its elusive metabolome and subtle transcriptome regulating its various stages of growth and development. Herein, we carried out an integrated metabolomic and transcriptomic profiling for different sized beech mushrooms across spatial components (cap and stipe) to delineate their signature pathways. We observed that metabolite profiles and differentially expressed gene (DEGs) displayed marked synergy for specific signature pathways according to mushroom sizes. Notably, the amino acid, nucleotide, and terpenoid metabolism-related metabolites and genes were more abundant in small-sized mushrooms. On the other hand, the relative levels of carbohydrates and TCA intermediate metabolites as well as corresponding genes were linearly increased with mushroom size. However, the composition of flavor-related metabolites was varying in different sized beech mushrooms. Our study explores the signature pathways associated with growth, development, nutritional, functional and organoleptic properties of different sized beech mushrooms.

## 1. Introduction

Mushrooms have traditionally been revered for their distinctive umami taste, flavors (earthy, meaty, or woodsy), and chewy texture coupled with varying nutraceutical functions [1]. Nutritionally, mushrooms are the quality source of proteins, polysaccharides, dietary fibers, vitamins, and minerals [2]. Beech mushrooms (*Hypsizygus marmoreus*) are native to East Asia and used in various culinary gourmets involving the ‘*Shimeji*’ mushroom groups [3]. In recent years, the applications of various strain improvement and cultivation methods have considerably improved the organoleptic properties of beech mushrooms attaining its ameliorated bitter taste, unique crunchy texture, and mild nutty flavor in a short cultivation period [4,5]. Beech mushrooms are rich in natural antioxidants and characteristic bioactive compounds, especially the hypsiziprenols having in vitro anti-proliferative effects on renal cancer cell lines [3,6]. Beech mushrooms have been increasingly recognized for their unique organoleptic, nutritional, and health promoting properties, however, the molecular mechanisms maneuvering the spatial distribution of nutrients and functional bio-actives across the fruiting body are largely unknown.

Mass spectrometry (MS)-based metabolomics approaches are widely used to study the metabolite profiles indicative of the key biosynthetic pathways governing the temporal growth and development of plants [7,8,9]. However, a limited number of metabolomics studies have reported the metabolic disparity among mushrooms across spatial dimensions and cultivars [3,10]. An approach utilizing integrated metabolomics and transcriptomics could provide a more powerful tool to understand the intertwined metabolism and molecular mechanisms of gene regulation through the construction of a single pathway [11,12,13,14]. Indeed, a number of studies have examined various microbes and plants, including *Arabidopsis*, *Salvia*, and *Penicillium* species using an integrated metabolomics–transcriptomics approach to reveal mechanisms underlying the biosynthesis of functional bio-actives including flavonoids, polyketides, and terpenoids [11,12,13,14].

Previously, we have reported the subtle variations in metabolite profiles of white and brown beech mushrooms across the stipe and cap portions which may help in mushroom selection and strain improvement [3]. In an extension to our previous study, we examined the metabolomic disparity among the beech mushroom groups of three different sizes (small, medium, and large) through the systematic construction of an integrated metabolic pathway derived from untargeted metabolite profiles and corresponding gene expression data. In the present study, we argue that different sized mushrooms may have markedly varying nutritional and functional values maneuvered by their distinct metabolomes, biosynthetic pathways, and associated gene expressions.

## 2. Results

### 2.1. Morphological Features and β-Glucan Levels for Different Sized Beech Mushrooms

**Figure 1:**
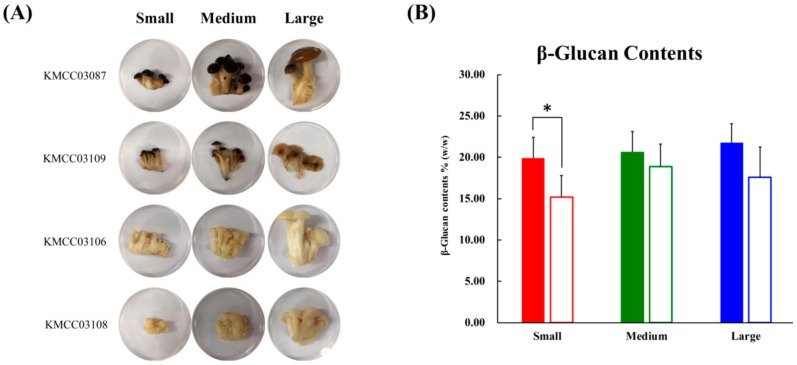
(**A**) Photographs of four beech mushrooms of different sizes. Developmental processes were divided according to three sizes: Small size; medium size; large size. (**B**) The graph represents the average β-glucan content. Data are represented as means ± S.D, with three biological replicates (*n* = 3) maintained for each of the four mushroom strains used in the study. Significant differences between the cap and stipe were identified by *t*-test (* *p*-value < 0.05). ■, cap parts; □, stipe parts.

The morphological aspects and different sizes (small, medium, and large) of the beech mushroom strains analyzed in this study are displayed in Figure 1A. The diameter of the caps and length of the stipes from the beech mushroom samples were measured, and they exhibited a linearly increasing trend with size (Table S1). The content of β-glucans in the cap were higher than those in the stipe samples, and these levels were proportionally increased with size with not significantly different. However, the β-glucan contents of the stipe decreased after the initial observed increase from small to medium sizes with no significance (Figure 1B).

### 2.2. Comprehensive Metabolite Profiles and Related Differentially Expressed Gene

#### 2.1.1. Primary and Secondary Metabolites

**Figure 2:**
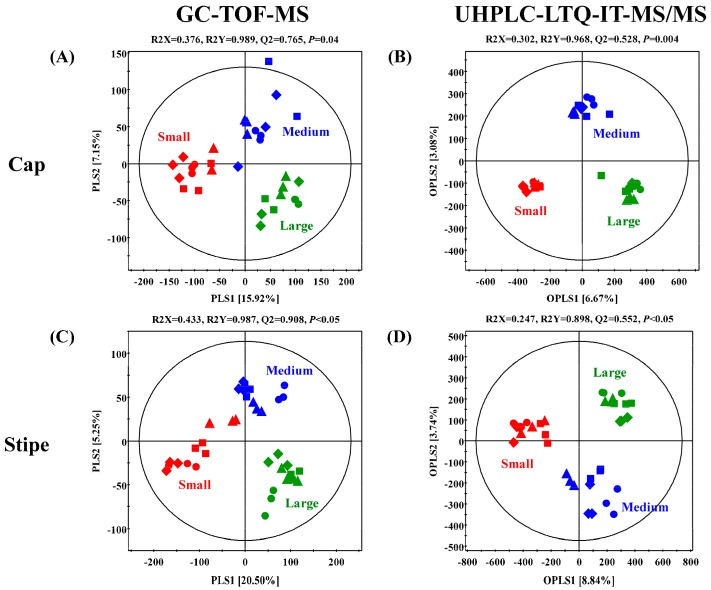
Partial least squares discriminant analysis (PLS-DA) and orthogonal partial least-discriminant analysis (OPLS-DA) score plots of caps (**A**,**B**) and stipes (**C**,**D**) collected, depending on different sizes of brown and white beech mushrooms, analyzed by GC-TOF-MS (**A**,**C**) and UHPLC-LTQ-IT-MS/MS (B and D). The biological replicates (*n* = 3) of four strain were analyzed by GC-TOF-MS and UHPLC-LTQ-IT-MS/MS. Small size is indicated by red color, medium size is indicated by blue color, and large size is indicated by green color. ▲: KMCC03087, ●: KMCC03109, ■: KMCC03106, and ◆: KMCC03108.

The GC-TOF-MS derived primary metabolite datasets for the caps and stipes of different sized (small, medium, and large) beech mushrooms were subjected to multivariate analyses incorporating the unsupervised PCA and the supervised PLS-DA. As shown in Figure 2A,C, the datasets for cap and stipe of small-sized mushroom samples were clustered apart from medium and large sized samples across PLS1 (15.92% and 20.50%), in respective score plots. On the other hand, both parts (cap and stipe) of medium-sized mushroom samples were segregated from small and large sizes along PLS2 (7.15% and 5.25%), in respective plots. We observed a similar consistency for the data patterns in PCA score plots (Figure S1A,C). Significantly different primary metabolites were identified and selected using variable importance in the projection value (VIP > 0.7) from PLS-DA models and *p*-value (*p* < 0.05) from one-way ANOVA. A total of 43 primary metabolites including 20 amino acids, one fatty acid, six organic acids, seven carbohydrates, four nucleotides, and five miscellaneous metabolites were characterized based on their retention times and mass fragment patterns data retrieved from National Institute of Standards and Technology (NIST, version 2.0., Gaithersburg, MD), Human metabolome Database (HMDB; http://www.hmdb.ca/), Wiley 9 and reference standards (Table 1 and Table 2). We scored a level 2 confidence for the identified primary metabolites and a level 3 confidence for non-identified metabolites identified in the present study based on the annotation criteria suggested by the Chemical Analysis Working Group of the Metabolomics Standards Initiative [15]. The relative levels of primary metabolites among different sized beech mushrooms in both parts (cap and stipe) were expressed in a heat map scale and translated into fold-change values (Table 1). Considering the metabolite profiles for the cap portions, the relative abundance of 16 amino acids (except for glutamic acid, ornithine, and cystathionine), 2 nucleobases, and N.I. 1 were significantly higher in the small-sized beech mushrooms than those from medium- or large-sized samples. Conversely, three organic acids, four carbohydrates, adenosine-diphosphate (ADP), and four miscellaneous metabolites (benzoic acid, ethanolamine, urea, and acetyl-glucosamine) were significantly higher in the cap portions of the medium- and large-sized beech mushroom samples. Intriguingly, the pattern of relative abundance of significantly discriminant metabolites for the stipe portion of the mushrooms of different sizes was similar to that of the cap portion. Notably, the relative abundance of 16 amino acids (with the exception of ornithine and cystathionine), three nucleobases, glutaric acid, citric acid, acetyl-glucosamine, and N.I. One was higher in the small-sized mushroom stipes. On the other hand, three organic acids, five carbohydrates, one lipid, and benzoic acid were abundant in the stipe portions of the medium- and large-sized mushrooms.

The UHPLC-LTQ-IT-MS/MS datasets from negative ion mode were utilized to examine the disparity in secondary metabolites profiles of cap and stipe portions from different sized beech mushrooms. The orthogonal partial least squares discriminant analysis (OPLS-DA) score plots significantly indicated clear grouping patterns in both parts (cap and stipe) among the different sized mushrooms (Figure 2B,D). Based on OPLS-DA score plots, the datasets for both parts (cap and stipe) of small-sized mushroom samples were clearly segregated from medium and large sized samples across OPLS 1 (6.67% and 8.84%) in respective plots. Further, the datasets for medium sized samples were clustered separately from small and large sized samples across OPLS 2 (3.08% and 3.74%) in corresponding score plots. Unlike the primary metabolites profile based on GC-TOF-MS datasets, the PCA score plots for secondary metabolites did not indicate any differences among the mushroom samples of different sizes (Figure S1B,D). A total of nine secondary metabolites, including two dicarboxylic acids, 3 hypsiziprenols and four non-identified (N.I.) ones were selected as the significantly discriminant metabolites in cap and stipe portions among the different sized beech mushrooms at VIP > 0.7 and *p*-value < 0.05 based on OPLS-DA models (Table 1). These secondary metabolites were putatively identified based on retention time, MS/MS fragment pattern, standard compounds, and published studies with level 2 confidence of annotation, while the non-identified metabolites scored by level 3 [15]. The relative levels of discriminant metabolites were displayed in a heat map scale (Table 1 and Table 2).

#### 2.1.2. Significantly Ciscriminant Metabolites-Related Differentially Expressed Genes

One-way ANOVA (*p*-value < 0.05) for RNA-sequencing data indicated 38 differentially expressed genes (DEGs) representing eight different metabolic pathways in different sized mushroom samples. Categorically, we observed different number of DEGs linked to the metabolism of carbohydrates (11), amino acids (7), nucleotides (4), terpenoids (4), lipids (3), xenobiotics biodegradation (3), other amino acids (2), and some miscellaneous metabolites (4). The DEGs representing various metabolic pathways for beech mushroom samples are listed in Table 3 with corresponding gene IDs, annotations, and enzyme commission (EC) numbers retrieved from the Kyoto Encyclopedia of Genes and Genomes (KEGG) pathway database.

The heat map data (Table 3) displays the linear increase in the expression levels of DEGs linked with the carbohydrate metabolism in beech mushroom according to size irrespective of the spatial components (cap and stipe) except for β-glucuronidase (EC 3.2.1.31), FAD-linked oxidoreductase (EC 1.1.3.15), laccase-1 (EC 1.10.3.2), and NADP-dependent malic enzyme (EC 1.1.1.40). Similarly, DEGs associated with xenobiotics biodegradation metabolism were highly expressed in medium and large sized mushrooms except for 3-hydroxy-phenylacetate 6-hydroxylase (EC 1.14.13.63). In contrast, seven amino acids, four nucleotides, four terpenoids, three lipids, and two other amino acid metabolism-related genes were lower expressed with increase in mushroom sizes notwithstanding their spatial variations. However, DEGs for meiotically up-regulated gene 158 protein (EC 2.1.1.44 and 1.14.99.51) displayed higher abundance in large sized (cap and stipe) mushrooms, while those for lipase 4 (EC 3.1.1.3) were abundant in cap (large sized) and stipe (small sized) mushrooms. Further, the relative abundance of DEGs associated with carbonic anhydrase (EC 4.2.1.1) in nitrogen metabolism and 4-coumarate CoA ligase (EC 6.2.1.12) in phenylpropanoid biosynthesis were relatively higher expressed in large sized mushrooms; whereas, phenylalanine-tRNA ligase (EC 6.1.1.20) in aminoacyl-tRNA biosynthesis and ferric reductase transmembrane component 5 (EC 1.16.1.7) in ferric chelate reduction were higher expressed in small-sized beech mushrooms.

### 2.3. Integrated Pathway Mapping for Discriminant Metabolites and DEGs in Different Sized Beech Mushrooms

**Figure 3:**
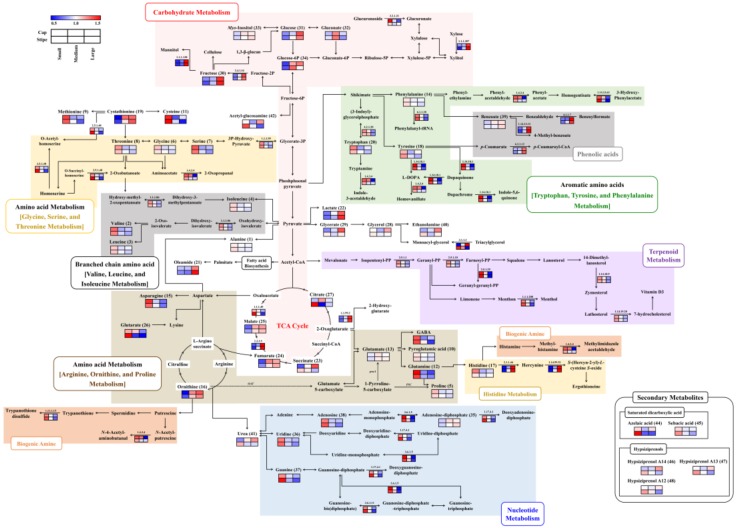
Constructed metabolic pathway that integrated relative metabolite contents and gene expression for three different sizes (small, medium and large) of beech mushrooms. The pathway was modified from the KEGG database (http://www.genome.jp/kegg/). The colored squares (blue-to-red) represent fold changes normalized by the average of each metabolite and gene expression level. EC numbers for the enzymes are listed in Table 3.

The synergies in the relative abundance of metabolites and associated DEGs among the different sized beech mushroom samples were visualized in corresponding pathway maps (Figure 3). Notably, several metabolites and their corresponding DEGs exhibited metabolite-gene correlation depending on the different mushroom sizes. The relative abundance of metabolites and associated genes were well-correlated with each other, especially those involved in valine, leucine, and isoleucine biosyntheses mediated by ketol acid reductoisomerase (EC 1.1.1.86); cysteine and methionine biosyntheses mediated by cystathionine gamma-synthase (EC 2.5.1.48); tryptophan by tryptophan synthase (EC 4.2.1.20); tyrosine by tyrosinase (EC 1.14.18.1); glycine, serine, and threonine by D-3-phosphoglycerate dehydrogenase (EC 1.1.1.95); malic acid by malate synthase (EC 2.3.3.9), β-glucans by β-glucan synthesis-associated protein (KPE6) and 1, 3-β-glucan synthase (EC 2.4.1.13); nucleotides (uridine, guanine, and adenosine) by ribonucleoside-diphosphate reductases (EC 1.17.4.1), exopolyphosphatase (EC 3.6.1.11) and Golgi apyrase (EC 3.6.1.5); phenolic acids such as benzoic acid with 4-coumarate CoA ligase-like 7 (EC 6.2.1.12), benzoylformate decarboxylase (EC 4.1.1.7) and benzoate 4-monooxygenase (EC 1.14.13.12).

Categorically, in the case of small-sized beech mushrooms, most of the amino acids, nucleotides, terpenoids, steroids, and biogenic amines were significantly abundant with higher expression of corresponding genes for both the cap and stipe portions (Figure 3 and Table 1 and Table 3). In particular, the levels of cysteine, glutamine, and asparagine were approximately 1.5–2-fold higher. The amino acid metabolism-related DEGs displayed a pattern similar to that of the metabolites themselves, but the histidine-derived ergothioneine-related gene, which is a meiotically up-regulated gene 158 protein (EC 2.1.1.44 and 1.14.99.51), exhibited the opposite pattern where it was highly expressed in the medium- and large-sized mushrooms. In case of nucleotide metabolism, levels of uridine, guanine, and adenosine decreased according to the size of the mushrooms, while adenosine-diphosphate levels increased. In congruence, the higher mRNA abundance was evident in both the cap and stipe of small-sized mushrooms. Intriguingly, the terpenoid, steroid-, and biogenic amine-related genes were highly expressed in small-sized mushrooms, but their corresponding metabolites were not detected in our data.

In the medium and large-sized mushrooms, most of the carbohydrates and phenolic acid derived metabolites and corresponding DEGs were significantly higher. Notably, the relative abundance of fructose and glucose were significantly higher than those observed in both parts of the small-sized mushrooms. Further, their related genes, except for β-glucuronidase (EC 3.2.1.31), were approximately 1.5–2-fold higher compared to their expression levels in both parts of the small-sized samples. Additionally, β-glucan-related genes, including putative β-glucan synthesis-associated protein (KPE6) and 1, 3-β-glucan synthase (EC 2.4.1.13), showed positive correlation with β-glucan contents, and this pattern considerably increased in both parts with increasing mushroom sizes (Figure 1B). Considering the metabolite intermediates of TCA cycle, the relative abundance of lactic acid, fumaric acid, and malic acid in both parts of medium- and large-sized beech mushrooms were higher. However, the citric acid and glutaric acid levels in the stipe portion of the small-sized mushrooms were significantly higher than medium and large-sized samples. Further, the phenolic acid-derived metabolites, such as benzoic acid and associated genes including benzoylformate decarboxylase (EC 4.1.1.7), benzoate 4-monooxygenase (EC 1.14.13.12), and 4-coumarate CoA ligase-like 7 (EC 6.2.1.12), were synergistically higher expressed in the medium and large sized mushrooms. In contrast, the levels of certain metabolites exhibited different patterns between the cap and stipe portions in different sized mushrooms. Most notably, the relative abundance of glyceric acid, glycerol, and ethanolamine, and the associated transcript for lipase 4 (EC 3.1.1.3) regulating the glycerolipid metabolism were higher in medium and large-sized mushroom caps, unlike the stipe portions. Additionally, succinic acid and some miscellaneous metabolites including urea, acetyl-glucosamine and hypiziprenols also displayed varying levels depending on different mushroom sizes across their spatial parts (cap and stipe).

## 3. Discussion

Considering the nutritional aspects, beech mushrooms are composed of various amino acids, organic acids, carbohydrates, nucleotides, and secondary metabolites, including hypsiziprenols and terpenoids (Figure 3). The untargeted metabolomic and transcriptomic analyses unraveled the compositional disparity among the different sized beech mushroom (small, medium, and large) samples having various implications.

In small-sized beech mushrooms (cap and stipe), most amino acids, nucleotides, terpenoids, and biogenic amines with corresponding DEGs displayed significantly higher abundance compared to medium- and large-sized mushrooms (Figure 3). Generally, amino acids are directly or indirectly related to the TCA cycle, glycolysis, and the urea cycle in an organism. Most amino acids are crucial metabolites that play an important function in fruiting body development and protein synthesis in mushrooms [16,17]. Considering the branched chain amino acid (BCAA) metabolism, higher relative levels of amino acids including valine, isoleucine, and leucine intertwined with higher expression of ketol-acid reductoisomerase (EC 1.1.1.86) were evident in small-sized beech mushrooms compared to medium- or large-sized analogs. BCAAs are crucial precursors for protein synthesis and, in particular, leucine is known to function in the regulation of intracellular signal transduction to control mRNA translation [17]. The serine component of glycine, serine, and threonine metabolism is not only the starting compound for the synthesis of various metabolites such as glycine, cysteine, and serine phospholipids, but is also used as a building block for protein synthesis [18]. The proline biosynthesis pathway from ornithine is also important in mushroom growth. Wagemaker et al. (2007) reported that ornithine can be converted into proline by ornithine aminotransferase (*OAT*) and pyrroline-5-carboxylate (*P5C*) and the mRNA of oat exhibit low levels during fruiting body developmental stages followed by a slight in transcription from post-harvest developmental stages of mushrooms [19]. In our study, gene expressions for *OAT* or *P5C* were not detected, however, the relative levels of proline were high in small-sized beech mushrooms whereas ornithine levels were high in medium and large-sized mushrooms. Proline plays a role in transporting nutrients from the mycelium to fruiting bodies depending on osmotic pressure. Reduction in proline levels prevents the constant outflow of resources from the mycelia to senescent sporocarps, whereas ornithine levels exhibit an increasing trend during growth [19]. Hence, we conjecture that differential expression of genes and metabolite profiles are complementary to the respective growth stages in mushroom development.

Regarding the nucleotide metabolism, the gene expression of ribonucleoside-diphosphate reductase (EC 1.17.4.1), putative exopolyphosphatase (EC 3.6.1.11) and Golgi apyrase (EC 3.6.1.5) were highly up-regulated in small-sized mushrooms. Additionally, metabolite levels of adenosine, guanine, and uridine except adenosine-diphosphate were relatively abundant in both parts (cap and stipe) of small-sized mushrooms compared to their relative levels in the medium- and large-sized mushrooms. Reportedly, nucleotide levels in immature mushrooms are higher than those in the mature mushrooms [20]. Nucleosides are precursor compounds for synthesizing genomic material, regulates reproductive functions, and also serve as energy source within the developing cells in mushrooms [20,21]. A similar research study indicated that the levels of nucleotides in the immature mushrooms were higher than those in the mature mushrooms [20].

Terpenoid metabolism involves conversion of farnesyl PP (FPP) to squalene, followed by its multistep enzymatic (farnesyl pyrophosphate synthase, geranylgeranyl diphosphate synthase, and short chain isoprenyl diphosphate synthase) conversion to lanosterol. Lanosterol is further metabolized into triterpenoids, which represent the basic structure of all steroids [22]. We observed the higher relative DEGs involved in terpenoid backbone biosynthesis (EC 2.5.1.1, 2.5.1.10, and 2.5.1.29) and steroid biosynthesis (EC 1.14.19.20 and 1.14.18.9) in small-sized beech mushrooms, however, the related metabolites were not detected in metabolomic profiling. Sterols are important for vegetative growth of fungi as they are the main components of membrane and precursors of steroid hormones regulating the sexual reproduction in mushrooms [22]. Hence, we argue that terpenoid metabolism may be one of the active metabolic pathways required at the early stage of beech mushroom development.

In the case of medium- and large-sized beech mushrooms, a higher abundance of the metabolites related to TCA cycle, carbohydrate metabolism, and phenolic acid metabolism coupled with higher gene levels were observed compared to those in small-sized mushrooms (Figure 3). Generally, the TCA cycle is a series of chemical reactions that occur through the oxidation of acetyl-CoA derived from carbohydrates, fats, and proteins to provide energy. Previously, it has been reported that the levels of organic acids including lactic acid, malic acid, and fumaric acid were higher in the mature stage of mushrooms [23]. The TCA cycle-related metabolites malic acid and fumaric acid perform energy storage functions during the development of the fruiting body [24]. In particular, malate synthase (*MS*) is the key enzyme of the TCA cycle, and this enzyme contributes to fruiting body formation and is up-regulated during fruiting body development [25,26]. Similarly, a higher abundance of carbohydrate derived metabolites including β-glucans, *myo*-inositol, glucose, fructose, and glucose-phosphate and their corresponding DEGs were observed for medium- and large-sized mushrooms. Functionally, carbohydrate metabolism influences a number of processes including glycolysis, the TCA cycle, hexose metabolism, and expansion of the fruiting-body in mushrooms besides generating the energy [10,27,28]. In particular, *myo*-inositol is a structural component of the cell membrane, while mannitol functions to promote expansion of the mushroom’s fruiting body with its levels heightened during the mature stages [29]. The bioactive phenolic compounds commonly reported in mushrooms are *p*-hydroxybenzoic, *p*-coumaric, and cinnamic acid which protect mushrooms against environmental and oxidative stress [30]. Previously, higher levels of total phenolic contents and several phenolic compounds were reported for matured stage mushrooms [31,32]. In congruence, we substantiated the higher levels of DEGs encoding benzoylformate decarboxylase (EC 4.1.1.7), benzoate 4-monooxygenase (EC 1.14.13.12) and 4-coumarate CoA ligase-like 7 (EC 6.2.1.12), in large-sized beech mushrooms representing the later stages of development.

Delineating the metabolomic-transcriptomic data across the cap and stipe portions of beech mushrooms, we observed a similar pattern with marginal disparity between spatial parts. Exceptionally, some metabolites including urea, glycerol, acetyl-glucosamine, and hepsiziprenols were differentially expressed in the different parts of beech mushrooms. Biochemically, urea is formed by purine degradation in ornithine cycle and functions the translocation of water and nutrients required for fruiting body development [33]. Wagemaker et al. (2006) reported the higher urease expression in stipe portion which gradually decrease during the later stages of mushroom development [34]. The mushrooms initially grow stipes and then grow caps [34]. In agreement, we observed that glycerol and acetyl-glucosamine, which are the backbone of cell membranes and cell walls, display relatively higher abundance in the stipes (small-sized) and caps (large-sized) of beech mushrooms. Additionally, hypsiziprenols have been poorly characterized in terms of their biosynthetic pathway, but given the general characteristics of secondary metabolites, hypsiziprenols are expected to crucial function in beech mushroom essential for survival and growth in competitive environments [3].

The commercial value of mushroom depends upon its organoleptic and sensory characteristics which in turn maneuvered by its blend of non-volatile and volatile metabolite components. Non-volatile tastant compounds in beech mushrooms mainly include amino acids, carbohydrates, and nucleotides. The hydrophobic amino acids such as proline, glycine, alanine, valine, leucine, tyrosine, and phenylalanine mainly impart the characteristic bitter taste [35]. In particular, proline is a major contributor to the bitter taste as it structurally interacts with respective retronasal receptors [35]. Conversely, glutamic acid, which belongs to the family of mono-sodium glutamate (MSG)-like amino acids, is a well-known contributor to umami taste [35]. In addition, certain nucleotides including 5′-nucleotides (5′-GMP, 5′-IMP, and 5′-GMP), contribute to the umami taste in mushroom [20]. Sugars and sugar alcohols, such as fructose and glucose, are also known to affect sweetness [10,36]. Considering the gustatory properties of metabolites, the small-sized beech mushrooms with higher relative abundance of most amino acids and nucleotides might be characterized to have more bitter-umami taste. On the other hand, the higher glutamic acid and carbohydrate sugar contents in large sized mushrooms may affect the more sweet-umami and low bitter taste.

In conclusion, integrated metabolomic and transcriptomic analyses of beech mushroom revealed discriminant metabolic states for its three-different sized (small, medium, and large) samples. Based on our data, we argued that beech mushrooms develop fruiting bodies from mycelium through up-regulating the amino acid, nucleotide, and terpenoid metabolism when they are small or at early stage of development. However, the later stages of fruiting body development involve an up-regulation of carbohydrate metabolism and the TCA cycle. The disparity of metabolite compositions in different sized beech mushrooms may significantly influence their nutritional, functional, gustatory, and sensory characteristics. Our results may aid in delineating the growth process, nutrient composition, and efficient cultivation periods for beech mushrooms based on their subtle metabolite and gene expression profiles.

## 4. Materials and Methods

### 4.1. Chemicals and Reagents

Methanol, acetonitrile, water, and hexane were purchased from Fisher Scientific (Pittsburgh, PA, USA). Methoxyamine hydrochloride, pyridine, *N*-methyl-*N*-(trimethylsilyl)-trifluoroacetamide (MSTFA), glacial acetic acid, and sulfuric acid were obtained from Sigma Chemical Co. (St. Louis, MO, USA). Sodium hydroxide and potassium hydroxide were purchased from Junsei Chemical (Tokyo, Japan). All enzymes and reagent buffers used were purchased from Megazyme Ltd. (Wicklow, Ireland).

### 4.2. Mushroom Materials

Brown (KMCC03087 and KMCC03109) and white (KMCC03106, and KMCC03108) beech mushroom fruiting bodies were provided by Bumwoo mushroom farm (Yeoju-si, Gyeonggi-do, Korea). The samples were cultivated using bottle technology for about three months, with four bottles of each strains were procured. Photographs of the fruiting body samples are shown in Figure 1B. The beech mushroom samples were randomly classified based morphology into three different sizes namely small, medium, and large. According to the diameter of cap portions, mushrooms were classified into small (< 1.0 cm), medium (between 1.0 and 2.0 cm), and large (> 2.0 cm), respectively. Growth characteristics of beech mushroom samples of different sizes are described in Table S1. Further, the fruiting bodies were separated into the cap and stipe portions prior to analyses. Three biological replications of four strains were used in this study.

### 4.3. Sample Preparation for Metabolite Profiling

The fruiting body samples were ground using liquid nitrogen and immediately placed in microfuge tubes. The number of fruiting bodies (cap and stipe portions) used for grinding were 10, 5, and 2 for small, medium, and large sizes, respectively. Ground samples (1 g) were extracted with 1 mL of 80% methanol by sonication for 5 min at 4 °C (Hettich Zentrifugen Universal 320, Tuttlingen, Germany) and then homogenized using a Retsch MM400 Mixer Mill (Retsch GmbH and Co., Haan, Germany) at 30 Hz for 10 min. After centrifugation for 10 min at 12,000 rpm and 4 °C (Gyrozen 1730R, Gyrozen Inc., Daejeon, Korea), the supernatants were filtered through a 0.2 μm polytetrafluoroethylene (PTFE) filter (Chromdisc, Daegu, Korea) and completely dried using a speed-vacuum concentrator (Biotron, Seoul, Korea). Dried extracts were re-dissolved in 80% methanol to a final concentration of 10 mg/mL for gas chromatography-time of flight-mass spectrometry (GC-TOF-MS) and ultrahigh performance liquid chromatography-linear trap quadruple-ion trap-mass spectrometry/mass spectrometry (UHPLC-LTQ-IT-MS/MS) analyses. For GC-TOF-MS analysis, the re-dissolved samples were completely dried again using a speed-vacuum concentrator prior to derivatization step. First, the dried samples were oximated with 50 µL of methoxyamine hydrochloride (20 mg/mL in pyridine) at 30 °C for 90 min. Next, 50 µL of the MSTFA was added to the samples for 30 min at 37 °C.

### 4.4. RNA Isolation for Transcriptome Analyis

RNA extraction was performed according to previous study [37]. The number of different-sized mushroom portions (cap and stipe) and biological replicates (n = 3) used for RNA extraction was the same as for metabolite extraction. The fruiting body of beech mushrooms were homogenized in liquid nitrogen with a mortar and pestle. The resulting homogenate was aliquoted to 1 g in Eppendorf-tubes and stored at –70 °C. Total RNA was extracted from each sample of the leaves separately using an RNeasy plant mini Kit (Qiagen, Valencia, CA, USA) according to the manufacturer’s instructions. RNA samples from *H. marmoreus* of different sizes were sequenced using an Illumina Hiseq2000 system, and 112,133,856 total reads were generated. The raw sequence data were extracted for the base pairs using the SolexaQA package. All sequence reads obtained from the samples of different sizes to optimize *de novo* assembly were used to assess *k*-mer sizes and assembled contigs. To select the best *de novo* assembly, several hash lengths were considered. The transcript annotation of *H. marmoreus* was defined by de novo assembly and validated by comparison to amino acid sequences in NCBI NR fungi, Uniprotkb fungi, KOG, and the KEGG database using BLASTX (e-value ≤ 1×10^-10^). The reads for each sequence tag were mapped to the assembled transcripts using Bowtie 2 (Johns Hopkins University, Baltimore, MD, USA, http://bowtie-bio.sourceforge.net) software (mismatch ≤ 2 bp), and the number of mapped clean reads for each transcript were counted. Then, mapped clean reads were normalized using the *DESeq* library as an R/Bioconductor package. To select differentially expressed genes (DEGs) among each analyzed sample, a two-fold change method to identify differences in expression that were above two-fold and a binomial test to satisfy the FDR (*p*-value) as less than 0.01 were simultaneously applied and calculated via *DESeq*. The process of RNA sequence analysis (de novo assembly, ordering contigs, and annotation) was performed by SEEDERS Company (Daejeon, South Korea).

### 4.5. GC-TOF-MS Analysis and UHPLC-LTQ-IT-MS/MS Analysis

GC-TOF-MS analysis was performed according to our previous study [3]. An Agilent 7890 gas chromatography, coupled with an Agilent 7693 autosampler (Agilent, Santa Clara, CA, USA) and installed with a Pegasus high-throughput (HT)-TOF-MS (Leco Corp., St. Joseph, MI, USA) program, was used for GC-TOF-MS analysis. Metabolites were separated by an Rtx-5MS column (30 m inner diameter × 0.25 mm i.d.; 0.25 mm particle size; Restek Corp. Bellefonte, PA, USA) was used with helium as the carrier gas at a constant flow rate of 1.5 mL/min. The GC oven temperature was maintained at 75 °C for 2 min, increased to 300 °C as final temperature over 15 min at rate of 15 °C/min. The front inlet and transfer line temperatures were 250 °C and 240 °C, respectively. The scanning mass range was 50–1000 m/z and the detector voltage was 1650 V. The derivatized samples (1 μL) were injected into a GC-TOF-MS instrument under split ratio (1:5). UHPLC-LTQ-IT-MS/MS was performed according to the method adapted from Park et al. [3] on the LTQ-XL mass spectrometry installed with ion trap-electrospray interface (Thermo Fisher Scientific, San José, CA) coupled with RS Autosampler, DIONEX UltiMate 3000 RS Pump, RS Column Compartment and RS Diode Array Detector (Dionex Corporation, Sunnyvale, CA, USA). Mushroom samples were separated on a Thermo Scientific Syncronis C18 UHPLC column with a 1.7 μm particle size. The flow rate was maintained at 0.3 mL/min with a 10 μL injection volume. The mobile phase consisted of Solvent A (0.1% formic acid in water) and Solvent B (0.1% formic acid in acetonitrile), the gradient flow was programmed as follow: The initial solvent condition was 10% of solvent B; the gradient was steadily increased from 10% solvent B to 100% solvent B over 18 min. Then, decreased to the initial conditions (10% solvent B) over 4 min, completing the total run time of 22 min. Mass spectra were obtained by electrospray ionization in positive and negative ion mode within a mass range of 150–1000 m/z. The photodiode array detector was programed at a wavelength range of 200–600 nm for detection and managed by 3D field. The operational parameters were as follows: Source voltage, ±5 kV; capillary voltage, 39 V; capillary temperature, 275 °C. Tandem MS analysis was carried out scan-type turbo data-dependent scanning under the conditions used for MS scanning. The analytical samples were randomly analyzed in blocks of ten runs followed by intermitted quality control (QC) samples to reduce systematic errors.

### 4.6. Measurement of β-Glucan Contents

The β-glucan contents were determined using a glucan assay kit (Megazyme Ltd., Wicklow, Ireland) according to the manufacturer’s procedures and our previous study [3], with some modification. For measurement of the total (α- and β-) glucan contents, the 24 dried beech mushroom samples (90 mg) were milled using a mortar and pestle, and 2 mL of cold 12 M sulfuric acid was added. Then, 10 mL of distilled water was added to each sample, and the samples were incubated in a boiling water bath at 100 °C for 2 h. After boiling, the samples were neutralized with 10M KOH, where 200 mM (pH 5.0) was added for a total volume of 100 mL. After centrifugation (1500 × g) for 15 min at 4 °C (Universal 320 R, Hettich, Zentrifugen, Germany), the reaction aliquots (100 μL) were mixed with 100 μL of exo-1,3-β-glucanase (20 U/mL) and β-glucosinase (4 U/mL) in 200 mM sodium acetate buffer (pH 5.0) and incubated at 40 °C for 1 h. Next, 3 mL of glucose determination reagent (GOPOD) was added and incubated at 40 °C for 20 min. The absorbance was recorded for the reacted samples at 510 nm.

For the determination of the α-glucan (starch and phytoglycogen) contents, the dried mushroom samples (100 mg) were dissolved in 2 mL of 2 M KOH and subsequently added to each sample with 8 mL of 1.2 M sodium acetate buffer (pH 3.8). Then, amyloglucosidase (1630 U/mL) and invertase (500 U/mL) were added to the solution and the samples were incubated at 40 °C for 20 min. After centrifugation (1500 × g) for 15 min at 4 °C (Universal 320 R, Hettich, Zentrifugen, Germany), 100 μL of the aliquot was mixed with 100 μL of 200 mM sodium acetate buffer (pH 5.0) and 3 mL of GOPOD reagent followed by an incubation at 40 °C for 20 min. The absorbance was recorded for the reacted samples at 510 nm. The levels of β-glucan were measured by subtracting α-glucan from the total glucan contents.

### 4.7. Data Processing and Multivariate Statistical Analysis

The GC-TOF-MS and UHPLC-LTQ-IT-MS/MS raw data files were converted to NetCDF (*.cdf) format using Leco ChromaTOF software (Version 4.44) and Thermo Xcalibur software (version 2.1, Thermo Fisher Scientific). After conversion, the alignments of NetCDF files were processed by the MetAlign software package (http://www.metalign.nl) to obtain data including retention times (min), normalized peak intensities, and corresponding mass (*m/z*). The MetAlign parameters were as follows: Peak slope factor, 2.0; peak threshold factor, 3.0; peak threshold, 10; average peak width at half height, 40, which correspond to a mass range of 50-1000 for GC-MS. In case of LC-MS, a peak threshold of 10 and average peak width at half height of 70 were set. The aligned data were applied to a treatment process for dealing with missing values and transformed to a data set for classification. The resulting data were exported to an Excel file, and statistical analysis was performed using SIMCA-P+ software (version 12.0, Umetrics, Umea, Sweden) to compare metabolites among samples. The data sets were auto-scaled (unit variance scaling), log-transformed and mean-centered in a column-wise fashion prior to principal component analysis (PCA) and partial least squares discriminant analysis (PLS-DA).

Principal component analysis (PCA), partial least-squares discriminant analysis (PLS-DA) and orthogonal partial least-squares discriminant analysis (OPLS-DA) were used to compare the different metabolites of the samples. Analysis of variance testing of cross-validated predictive residuals (CV-ANOVA) is a diagnostic tool for assessing the reliability of PLS and OPLS models applied by SIMCA-P+ version 12.0. CV-ANOVA uses a significance test (*F*-test) of the null hypothesis of equal residuals of the two compared models. The *F*-test, based on the ratio MS regression/MS residual, analyzes the significance of model [38]. The model of PLS-DA and OPLS-DA based on our MS data showed a significantly different model (*p*-value < 0.05). The significantly discriminant metabolites were selected based on the variable importance in the projection (VIP) values > 0.7 and tested for significance at *p*-value < 0.05. The selected metabolites obtained from GC-TOF-MS and UHPLC-LTQ-IT-MS/MS were tentatively identified based on various data comparing their retention time, mass spectrum, MS^n^ fragment, and UV spectrum data with those for standard compounds analyzed under identical condition and various available database including published papers [3], the National Institute of Standards and Technology (NIST) database (Version 2.0, 2011, FairCom, Gaithersburg, MD, USA), the Human Metabolome Database (HMDB; http://www.hmdb.ca/), Wiley 9, In house libraries, and Dictionary of Natural Product (CCD, Copyright 2008, Taylor and Francis Group, Boca Raton, FL, USA). Significant statistical differences including metabolome and RNA-sequencing data were determined by analysis of variance (ANOVA) coupled with Duncan’s multiple range tests using PASW statistica 18 (SPAA, Inc., Chicago, IL, USA) and visualized by heat map analysis using MEV software, version 4.8 (multiple array viewer).

## Figures and Tables

**Table 1:** List of metabolites identified in caps and stipes of beech mushrooms of three different sizes.

**GC-TOF-MS (Primary Metabolites)**							
**No. ^a^**	**Metabolite ^b^**		**Cap**			**Stipe**	
		**Small**	**Medium**	**Large**	**Small**	**Medium**	**Large**
***Amino acids***							
1	Alanine **	**1.03**	**0.99**	**0.98**	**1.06**	**0.97**	**0.97**
2	Valine	**1.21**	**0.87**	**0.91**	**1.19**	**0.94**	**0.87**
3	Leucine **	**1.07**	**0.95**	**0.98**	**1.09**	**0.98**	**0.93**
4	Isoleucine	**1.06**	**0.95**	**0.99**	**1.07**	**0.99**	**0.95**
5	Proline	**1.09**	**0.95**	**0.96**	**1.06**	**1.00**	**0.94**
6	Glycine	**1.08**	**0.94**	**0.97**	**1.04**	**1.01**	**0.95**
7	Serine	**1.23**	**0.86**	**0.90**	**1.22**	**0.95**	**0.83**
8	Threonine	**1.14**	**0.91**	**0.95**	**1.10**	**0.99**	**0.91**
9	Methionine	**1.26**	**0.86**	**0.88**	**1.27**	**0.99**	**0.74**
10	Pyroglutamic acid **	**1.04**	**1.00**	**0.96**	**1.07**	**0.98**	**0.95**
11	Cysteine	**1.31**	**0.73**	**0.96**	**1.42**	**0.95**	**0.64**
12	Glutamine *	**1.47**	**0.82**	**0.71**	**1.30**	**0.88**	**0.82**
13	Glutamine acid *	**0.96**	**1.04**	**0.99**	**0.95**	**1.03**	**1.03**
14	Phenylalanine	**1.04**	**0.98**	**0.99**	**1.06**	**1.00**	**0.94**
15	Asparagine	**1.29**	**0.93**	**0.78**	**1.24**	**0.98**	**0.78**
16	Ornithine	**0.60**	**1.16**	**1.24**	**0.73**	**1.08**	**1.19**
17	Histidine *	**1.18**	**0.88**	**0.94**	**1.04**	**0.96**	**1.00**
18	Tyrosine	**1.10**	**0.88**	**1.01**	**1.16**	**0.96**	**0.88**
19	Cystathionine	**0.59**	**1.20**	**1.21**	**0.69**	**1.04**	**1.27**
20	Tryptophan *	**1.17**	**0.86**	**0.97**	**1.11**	**0.99**	**0.90**
*Fatty acids*							
21	Oleamide **	**0.91**	**0.91**	**1.18**	**0.82**	**0.79**	**1.39**
*Organic acids*							
22	Lactic acid **	**0.79**	**0.99**	**1.22**	**0.77**	**0.89**	**1.34**
23	Succinic acid *	**0.73**	**1.02**	**1.25**	**1.08**	**1.03**	**0.89**
24	Fumaric acid	**0.71**	**1.17**	**1.12**	**0.83**	**1.04**	**1.13**
25	Malic acid	**0.75**	**1.11**	**1.14**	**0.85**	**1.05**	**1.10**
26	Glutaric acid **	**1.12**	**1.07**	**0.81**	**1.84**	**0.68**	**0.48**
27	Citric acid **	**0.90**	**1.14**	**0.96**	**1.47**	**0.60**	**0.93**
*Carbohydrates*							
28	Glycerol *	**1.00**	**0.88**	**1.12**	**1.03**	**0.98**	**1.00**
29	Glyceric acid *	**0.82**	**0.95**	**1.24**	**1.02**	**0.94**	**1.03**
30	Fructose **	**0.82**	**0.89**	**1.29**	**0.73**	**0.91**	**1.36**
31	Glucose **	**0.82**	**0.93**	**1.24**	**0.79**	**1.01**	**1.21**
32	Gluconic acid **	**0.90**	**1.16**	**0.94**	**0.95**	**1.22**	**0.83**
33	*myo*-Inositiol	**0.93**	**1.04**	**1.03**	**0.93**	**1.00**	**1.06**
34	Glucose 6-phosphate	**0.70**	**1.12**	**1.19**	**0.73**	**1.25**	**1.02**
*Nucleosides*							
35	Adenosine-diphosphate	**0.92**	**1.05**	**1.03**	**0.93**	**1.00**	**1.06**
36	Uridine **	**1.16**	**0.96**	**0.88**	**1.38**	**0.89**	**0.73**
37	Guanine	**1.18**	**0.93**	**0.90**	**1.24**	**0.94**	**0.82**
38	Adenosine	**1.18**	**0.92**	**0.90**	**1.27**	**0.93**	**0.80**
*Miscellaneous*							
39	Benzoic acid	**0.92**	**1.03**	**1.05**	**0.96**	**0.97**	**1.06**
40	Ethanolamine *	**0.87**	**0.95**	**1.18**	**1.07**	**0.91**	**1.02**
41	Urea *	**0.82**	**1.03**	**1.15**	**1.12**	**0.94**	**0.94**
42	Acetyl-glucosamine	**1.01**	**0.85**	**1.14**	**1.29**	**0.97**	**0.74**
43	N.I 1	**1.15**	**1.08**	**0.77**	**1.35**	**0.94**	**0.71**
**UHPLC-LTQ-IT-MS/MS (Secondary metabolites)**							
**No. ^a^**	**Metabolite ^c^**		**Cap**			**Stipe**	
		**Small**	**Medium**	**Large**	**Small**	**Medium**	**Large**
*Saturated dicarboxylic acids*							
44	Azelaic acid *	**1.07**	**0.87**	**1.06**	**1.67**	**0.68**	**0.65**
45	Sebacic acid **	**1.07**	**0.96**	**0.98**	**1.22**	**0.96**	**0.82**
***Hypsiziprenols***							
46	Hypsiziprenol A14 *	**0.90**	**0.92**	**1.18**	**1.13**	**1.00**	**0.87**
47	Hypsiziprenol A13 **	**0.99**	**0.92**	**1.09**	**1.21**	**0.99**	**0.80**
48	Hypsiziprenol A12 **	**0.99**	**0.97**	**1.04**	**1.19**	**1.04**	**0.77**
*Non-identifications*							
49	N.I. 2 **	**1.02**	**1.07**	**0.91**	**1.22**	**1.19**	**0.59**
50	N.I. 3	**0.62**	**0.87**	**1.51**	**0.56**	**0.67**	**1.77**
51	N.I. 4 *	**0.82**	**0.95**	**1.23**	**1.03**	**1.07**	**0.90**
52	N.I. 5 **	**1.04**	**0.94**	**1.02**	**1.19**	**0.97**	**0.84**

^a^ Number of metabolites.; ^b^ Selected and identified primary metabolites based on variable importance in projection (VIP) value (>0.7) and *p*-value (<0.05) in both VIP1 and VIP2 by PLS-DA.; ^c^ Selected and identified secondary metabolites based on variable importance in projection (VIP) value (>0.7) and *p*-value (<0.05) in both VIP1 and VIP2 by OPLS-DA.;* Only selected metabolites in caps.; ** Only selected metabolites in stipes.; The colored squares (blue-to-red) represent fold changes normalized by average of each metabolites of beech mushroom. The color scheme is as follows: Lower limit value, 0 (blue); middle limit value, 1 (white); upper limit value, 1.5 (red).

**Table 2:** Tentative identified primary and secondary metabolites in caps and stipes of beech mushrooms of three different sizes based on GC-TOF-MS and UHPLC-LTQ-IT-MS/MS.

GC-TOF-MS						UHPLC-LTQ-IT-MS/MS							
No. ^a^	Metabolite ^b^	Rt ^c^_(min)_	Mass Fragment Pattern (*m/z*)	TMS ^d^	ID	No. ^a^	Metabolite ^e^	Rt ^c^_(min)_	[M-H]^-^	[M+H]^+^	M.W.	MS ^n^ [M-H]- Fragment Pattern	ID
***Amino acids***						***Saturated dicarboxylic acids***							
1	Alanine **	5.15	100, 103, 116, 190	(TMS)_2_	STD/MS	44	Azelaic acid *	8.36	187	–	188	187>125>97	STD
2	Valine	6.64	45, 59, 100, 144, 218	(TMS)_2_	STD/MS	45	Sebacic acid **	9.40	201	–	202	201>183, 139	STD
3	Leucine **	7.20	73, 100, 102, 133, 158	(TMS)_2_	STD/MS	***Hypsiziprenols***							
4	Isoleucine	7.42	59, 73, 100, 158, 218	(TMS)_2_	STD/MS	46	Hypsiziprenol A14 *	14.42	1214	1170	1169	1168>1098>1080, 1014	REF
5	Proline	7.47	59, 73, 142, 147	(TMS)_2_	STD/MS	47	Hypsiziprenol A13 **	14.48	1082	1084	1083	1082>300>882, 816	REF
6	Glycine	7.55	86, 100, 133, 174, 248	(TMS)_3_	STD/MS	48	Hypsiziprenol A12 **	14.56	996	998	997	996>814>956, 730	REF
7	Serine	8.05	73, 100, 147, 204, 218	(TMS)_3_	STD/MS	Non-identifications							
8	Threonine	8.31	73, 101, 117, 129, 219	(TMS)_3_	STD/MS	49	N.I. 2 **	6.22	492	494	493	494>474>254	–
9	Methionine	9.45	61, 100, 128, 147, 176	(TMS)_2_	STD/MS	50	N.I. 3	8.16	432	434	433	434>306>288, 272, 254	–
10	Pyroglutamic acid **	9.51	59, 147, 156, 230, 258	(TMS)_2_	STD/MS	51	N.I. 4 *	9.05	199	201	200	–	–
11	Cysteine	9.75	73, 100, 147, 218, 220	(TMS)_3_	STD/MS	52	N.I. 5 **	10.71	329	–	–	–	–
12	Glutamine *	10.07	128, 139, 147, 154, 227	(TMS)_3_	STD/MS								
13	Glutamine acid *	10.24	56, 84, 127, 156, 246	(TMS)_3_	STD/MS								
14	Phenylalanine	10.34	73, 100, 147, 192, 218	(TMS)_2_	STD/MS								
15	Asparagine	10.66	73, 116, 132, 141, 188	(TMS)_3_	STD/MS								
16	Ornithine	11.73	73, 142, 147, 174, 100	(TMS)_4_	STD/MS								
17	Histidine *	12.48	74, 100, 107, 154, 254	(TMS)_3_	STD/MS								
18	Tyrosine	12.57	73, 100, 147, 218	(TMS)_3_	STD/MS								
19	Cystathionine	14.26	73, 100, 128, 147, 218	(TMS)_4_	STD/MS								
20	Tryptophan *	14.36	73, 100, 147, 202, 218	(TMS)_3_	STD/MS								
***Fatty acids***													
21	Oleamide **	15.33	116, 128, 131, 144, 198	(TMS)_1_	STD/MS								
***Organic acids***													
22	Lactic acid **	4.99	59, 75, 117, 147, 191	(TMS)_2_	STD/MS								
23	Succinic acid *	7.58	55, 73, 129, 147, 247	(TMS)_2_	STD/MS								
24	Fumaric acid	7.83	75, 115, 143, 147, 245	(TMS)_2_	STD/MS								
25	Malic acid	9.19	55, 101, 133, 147, 233	(TMS)_3_	STD/MS								
26	Glutaric acid **	9.89	45, 55, 112, 156, 198	(TMS)_2_	STD/MS								
27	Citric acid **	11.76	45, 67, 73, 147, 273	(TMS)_4_	STD/MS								
***Carbohydrates***													
28	Glycerol *	7.23	75, 103, 117, 133, 205	(TMS)_3_	STD/MS								
29	Glyceric acid *	7.78	103, 117, 133, 189, 292	(TMS)_3_	STD/MS								
30	Fructose **	12.18	103, 117, 133, 217, 307	(TMS)_5_	STD/MS								
31	Glucose **	12.36	129, 133, 157, 160, 319	(TMS)_5_	STD/MS								
32	Gluconic acid **	13.06	103, 117, 205, 217, 292, 333	(TMS)_5_	STD/MS								
33	myo-Inositiol	13.66	103, 129, 147, 191, 217, 305	(TMS)_6_	STD/MS								
34	Glucose 6-phosphate	15.01	129, 160, 299, 387	(TMS)_6_	STD/MS								
***Nucleosides***													
35	Adenosine-diphosphate	7.26	133, 193, 207, 299, 300	(TMS)_3_	STD/MS								
36	Uridine **	15.63	45, 169, 217, 245	(TMS)_4_	STD/MS								
37	Guanine	13.78	99, 100, 131, 352	(TMS)_3_	STD/MS								
38	Adenosine	16.60	192, 217, 230, 236, 245	(TMS)_4_	STD/MS								
***Miscellaneous***													
39	Benzoic acid	6.96	51, 77, 105, 135, 179	(TMS)_2_	STD/MS								
40	Ethanolamine *	7.15	86, 100, 133, 147, 174	(TMS)_3_	STD/MS								
41	Urea *	7.20	45, 73, 147, 171, 189	(TMS)_2_	STD/MS								
42	Acetyl-glucosamine	13.58	87, 117, 129, 173, 202, 205	(TMS)_4_	STD/MS								
43	N.I 1	11.91	133, 147, 191, 217, 260	–	–								

^a^ Number of metabolites.; ^b^ Selected and tentatively identified primary metabolites based on variable importance in projection (VIP) value (>0.7) and *p*-value (<0.05) in both VIP1 and VIP2 by PLS-DA.; ^c^ Retention time.; ^d^ TMS, the number of trimethylsilyl groups; ^e^ Selected and tentatively identified secondary metabolites based on variable importance in projection (VIP) value (>0.7) and *p*-value (<0.05) in both VIP1 and VIP2 by OPLS-DA.; ^f^ Identification. STD/MS, comparing with standard compounds analyzed under same condition and mass spectra comparison with HMDB, NIST database, and Wiley 9.; ^g^ Identification. STD, comparing with standard compounds analyzed under same condition; REF, published paper ([3] Park et al., 2017); CCD, Dictionary of Natural product; ^h^ [H+HCOOH-H]^−^; * Only selected metabolites in caps (VIP > 0.7, *p* < 0.05).; ** Only selected metabolites in stipes (VIP > 0.7, *p* < 0.05).

**Table 3:** Differentially expressed genes (DEGs) related to metabolite biosyntheses in three different sized beech mushrooms.

No.	Gene ID ^a^	Sub-Classification	Annotation ^b^	EC number ^c^		Cap			Stipe	
					Small	Medium	Large	Small	Medium	Large
***Carbohydrate Metabolism***										
1	Htessellatus1SL000147t0011	Ascorbate and aldarate metabolism	Versatile peroxidase VPL1	1.11.1.13	**0.78**	**0.91**	**1.30**	**0.76**	**0.91**	**1.33**
2	Htessellatus1SL000266t0019	Butanoate metabolism	*L*-2-Hydroxyglutarate dehydrogenase	1.1.99.2	**0.67**	**0.91**	**1.43**	**0.63**	**0.96**	**1.41**
3	Htessellatus1SL008026t0008	Glyoxylate and dicarboxylate metabolism	Malate synthase	2.3.3.9	**0.61**	**0.77**	**1.61**	**0.50**	**0.96**	**1.54**
4	Htessellatus1SL013065t0001	Starch and sucrose metabolism	Putative *β*-glucan synthesis-associated protein	KPE6	**0.69**	**0.87**	**1.45**	**0.63**	**0.87**	**1.50**
5	Htessellatus1SL006041t0015	Starch and sucrose metabolism	1,3-*β*-glucan synthase	2.4.1.34	**0.69**	**0.95**	**1.36**	**0.69**	**1.03**	**1.28**
6	Htessellatus1SL008799t0004	Fructose and mannose metabolism	NADP-dependent mannitol dehydrogenase	1.1.1.138	**0.49**	**0.70**	**1.81**	**0.49**	**0.64**	**1.86**
7	Htessellatus1SL005645t0003	Pentose and glucuronate interconversions	Putative NAD(P)H-dependent D-xylose reductase	1.1.1.307	**0.77**	**0.83**	**1.40**	**0.47**	**0.89**	**1.65**
8	Htessellatus1SL007796t0013	Pentose and glucuronate interconversions	β-Glucuronidase	3.2.1.31	**1.48**	**0.94**	**0.58**	**1.09**	**0.90**	**1.02**
9	Htessellatus1SL004183t0003	Ascorbate and aldarate metabolism	Laccase-1	1.10.3.2	**1.26**	**1.03**	**0.71**	**1.15**	**0.96**	**0.89**
10	Htessellatus1SL000110t0005	Glyoxylate and dicarboxylate metabolism	FAD-linked oxidoreductase	1.1.3.15	**1.72**	**0.90**	**0.37**	**1.21**	**1.11**	**0.68**
11	Htessellatus1SL004210t0003	Pyruvate metabolism	NADP-dependent malic enzyme	1.1.1.40	**1.15**	**1.04**	**0.81**	**1.52**	**0.99**	**0.49**
***Amino acid metabolism***										
12	Htessellatus1SL006626t0002	Histidine metabolism	Meiotically up-regulated gene 158 protein	2.1.1.44 / 1.14.99.51	**0.41**	**0.78**	**1.80**	**0.43**	**0.96**	**1.61**
13	Htessellatus1SL007811t0003	Histidine metabolism	Putative bifunctional amine oxidase	1.4.3.4	**1.33**	**1.27**	**0.40**	**1.17**	**0.92**	**0.91**
14	Htessellatus1SL001236t0007	Cysteine and methionine metabolism	Putative cystathionine gamma-synthase	2.5.1.48	**1.21**	**0.94**	**0.85**	**1.49**	**0.97**	**0.54**
15	Htessellatus1SL005032t0006	Glycine, serine and threonine metabolism	D-3-phosphoglycerate dehydrogenase 1	1.1.1.95	**1.23**	**0.87**	**0.90**	**1.24**	**0.98**	**0.78**
16	Htessellatus1SL002183t0001	Phenylalanine, tyrosine and tryptophan biosynthesis	Tryptophan synthase	4.2.1.20	**1.23**	**1.03**	**0.74**	**1.13**	**1.13**	**0.74**
17	Htessellatus1SL009909t0005	Tyrosine metabolism	Tyrosinase	1.14.18.1	**1.77**	**0.88**	**0.35**	**1.37**	**1.01**	**0.62**
18	Htessellatus1SL006526t0001	Valine, leucine and isoleucine biosynthesis	Putative ketol acid reductoisomerase	1.1.1.86	**1.22**	**0.96**	**0.83**	**1.13**	**1.04**	**0.82**
***Nucleotide metabolism***										
19	Htessellatus1SL014146t0001	Pyrimidine metabolism	Ribonucleoside-diphosphate reductase small chain	1.17.4.1	**1.21**	**1.00**	**0.79**	**1.45**	**0.98**	**0.57**
20	Htessellatus1SL002783t0004	Pyrimidine & Purine metabolism	Ribonucleoside diphosphate reductase large chain	1.17.4.1	**1.19**	**1.02**	**0.79**	**1.36**	**1.04**	**0.60**
21	Htessellatus1SL006389t0001	Pyrimidine & Purine metabolism	Golgi apyrase	3.6.1.5	**1.57**	**1.02**	**0.42**	**1.40**	**1.10**	**0.50**
22	Htessellatus1SL012650t0011	Purine metabolism	Putative exopolyphosphatase	3.6.1.11	**1.24**	**1.01**	**0.75**	**1.44**	**1.03**	**0.53**
***Terpenoid metabolism***										
26	Htessellatus1SL002734t0005	Monoterpenoid biosynthesis	Neomenthol dehydrogenase	1.1.1.208	**1.25**	**0.98**	**0.77**	**1.37**	**1.06**	**0.57**
23	Htessellatus1SL007764t0004	Terpenoid backbone biosynthesis	Farnesyl pyrophosphate synthase	2.5.1.1 / 2.5.1.10	**1.23**	**0.97**	**0.8**	**1.12**	**1.04**	**0.84**
24	Htessellatus1SL001218t0008	Terpenoid backbone biosynthesis	Geranylgeranyl diphosphate synthase	2.5.1.29	**1.48**	**1.09**	**0.42**	**1.72**	**0.96**	**0.33**
25	Htessellatus1SL000034t0003	Terpenoid backbone biosynthesis	Short chain isoprenyl diphosphate synthase	2.5.1.1 / 2.5.1.10 / 2.5.1.29	**1.34**	**1.01**	**0.64**	**1.27**	**1.06**	**0.67**
***Lipid metabolism***										
27	Htessellatus1SL005093t0001	Glycerolipid metabolism	Lipase 4	3.1.1.3	**0.33**	**0.94**	**1.73**	**1.43**	**1.21**	**0.35**
28	Htessellatus1SL000281t0004	Steroid biosynthesis	Lathosterol oxidase	1.14.19.20	**1.19**	**0.99**	**0.83**	**1.20**	**0.96**	**0.85**
29	Htessellatus1SL000053t0001	Steroid biosynthesis	Methylsterol monooxygenase	1.14.18.9	**1.20**	**1.02**	**0.77**	**1.16**	**1.08**	**0.77**
***Xenobiotics biodegradation and metabolism***										
30	Htessellatus1SL003043t0001	Aminobenzoate degradation	Benzoylformate decarboxylase	4.1.1.7	**0.69**	**0.68**	**1.63**	**0.66**	**0.92**	**1.41**
31	Htessellatus1SL010123t0002	Benzoate degradation	Benzoate 4-monooxygenase	1.14.13.12	**0.67**	**0.91**	**1.42**	**0.54**	**0.90**	**1.57**
32	Htessellatus1SL006522t0004	Styrene degradation	3-Hydroxyphenylacetate 6-hydroxylase	1.14.13.63	**1.32**	**1.10**	**0.57**	**1.81**	**0.84**	**0.35**
***Other amino acid metabolism***										
34	Htessellatus1SL004301t0002	Glutathione metabolism	Peroxiredoxin	1.11.1.15	**1.23**	**0.99**	**0.78**	**1.36**	**1.01**	**0.64**
33	Htessellatus1SL000068t0001	Peptidylprolyl isomerase	FK506-binding protein 4	5.2.1.8	**1.32**	**0.95**	**0.73**	**1.35**	**1.01**	**0.64**
***Miscellaneous metabolism***										
36	Htessellatus1SL009373t0001	Nitrogen metabolism	Carbonic anhydrase	4.2.1.1	**0.75**	**0.80**	**1.45**	**0.56**	**0.83**	**1.61**
35	Htessellatus1SL006510t0001	Phenylpropanoid biosynthesis	4-Coumarate CoA ligase-like 7	6.2.1.12	**0.86**	**0.88**	**1.26**	**0.72**	**0.95**	**1.33**
37	Htessellatus1SL002357t0005	Aminoacyl-tRNA biosynthesis	Phenylalanine-tRNA ligase	6.1.1.20	**1.28**	**1.01**	**0.71**	**1.36**	**1.04**	**0.60**
38	Htessellatus1SL012836t0001	Ferric-chelate reductase	Ferric reductase transmembrane component 5	1.16.1.7	**1.38**	**1.13**	**0.49**	**1.25**	**1.14**	**0.61**

^a^ Gene number is approximately the same as *Hypsizygus marmoreus*, which suggests a similar matched gene number in mushroom family. Genes were selected by *p*-value < 0.05.; ^b^ Biological information in sequences.; ^c^ Enzyme commission number for enzyme from KEGG database (http://www.genome.jp/kegg/).; The colored squares (blue-to-red) represent fold changes normalized by the average of each gene expression of beech mushroom. The color scheme is as follows: Lower limit value, 0 (blue); middle limit value, 1 (white); upper limit value, 2 (red).

## Supplementary Materials

Supplementary materials can be found at https://www.mdpi.com/1422-0067/20/23/6007/s1.

## Abbreviations

GC-TOF-MS	Gas chromatography-time of flight-mass spectrometry
UHPLC-LTQ-IT-MS/MS	Ultrahigh performance liquid chromatography-linear trap quadruple-ion trap-mass spectrometry/mass spectrometry
PCA	Principal component analysis
PLS-DA	Partial least squares-discriminant analysis
OPLS-DA	Orthogonal partial least squares-discriminant analysis
DEG	Differentially expressed gene
EC	Enzyme commission
