# Supplementary material for: Integrated Metabolomics and Transcriptomics Unravel the Metabolic Pathway Variations for Different Sized Beech Mushrooms
Source: Int J Mol Sci. 2019 Nov 28;20(23):6007. doi: 10.3390/ijms20236007 (PMC6928633; doi:10.3390/ijms20236007)
Supplement: Supplementary file 1 [file ijms-20-06007-s001.pdf]

Integrated Metabolomics and Transcriptomics Unravel the Metabolic Pathway Variations for Different Sized Beech Mushrooms

Su Young Son [1], Yu Jin Park [1], Eun Sung Jung [2], Digar Singh [1], Young Wook Lee [3], Jeong-Gu Kim [4,*] and Choong Hwan Lee [1,2,5,*]

[1] Department of Bioscience and Biotechnology, Konkuk University, Seoul 05029, Republic of Korea; syson119@naver.com (S.Y.S.); y_zing@naver.com (Y.J.P.); singhdigar@gmail.com (D.S.)
[2] Department of Systems Biotechnology, Konkuk University, Seoul 05029, Republic of Korea; jes708@naver.com
[3] Bumwoo Mushroom Farm Co. Ltd., 538 Yeoyang 1-ro, Yeoju 12609, Republic of Korea; bumwoo0789@naver.com
[4] Genomics Division, National Academy of Agricultural Science, Rural Development Administration, Jeonju 54874, Republic of Korea
[5] Research Institute for Bioactive-Metabolome Network, Konkuk University, Seoul, 05029, Republic of Korea
* Correspondence: jkim5aug@korea.kr (J.-G.K.); Tel.: +82-63-238-45-66 (J.-G.K.); chlee123@konkuk.ac.kr (C.H.J.); Tel.: +82-2-2049-6177(C.H.J.)

Supplementary Table

Table S1. Growth characteristics of beech mushroom fruiting bodies of various size: (Small) cap diameter less than 1.0 cm, (Medium) cap diameter between 1.0 and 2.0 cm, (Large) cap diameter higher than 2.0 cm.

Strain	Size	Cap diameter (cm)	Stipe length (cm)
KMCC03087	Small	0.83 ± 0.21	1.33 ± 0.21
	Medium	1.40 ± 0.36	2.40 ± 0.17
	Large	3.43 ± 0.60	3.10 ± 0.66
KMCC03109	Small	0.67 ± 0.06	1.67 ± 0.15
	Medium	1.07 ± 0.12	2.93 ± 0.49
	Large	2.33 ± 0.15	2.97 ± 0.15
KMCC03106	Small	0.50 ± 0.10	1.47 ± 0.25
	Medium	1.07 ± 0.21	3.00 ± 0.50
	Large	2.40 ± 0.36	4.00 ± 0.50
KMCC03108	Small	0.73 ± 0.06	0.60 ± 0.10
	Medium	1.17 ± 0.29	2.17 ± 0.58
	Large	2.50 ± 0.50	2.23 ± 0.64

Table S2. Comparison between beech mushrooms of three different sized samples (caps and stipes) and standard compounds equally analyzed by GC-TOF-MS

Beech mushroom sample				Standard compounds		
No. [a]	Metabolites [b]	Ret (min) [c]	Mass fragment pattern (*m*/*z*)	Metabolites	Ret (min)	Mass fragment pattern (*m*/*z*)
1	Alanine	5.44	73, 100, 103, 116, 117, 147, 190	Alanine	5.44	73, 116, 147, 117, 59, 75, 190
2	Valine	6.64	45, 59, 73, 100, 144, 147, 218	Valine	6.63	73, 144, 218, 100, 59, 75, 147
3	Leucine	7.20	73, 100, 102, 133, 147, 158	Leucine	7.18	73, 158, 102, 59, 75, 159, 147
4	Isoleucine	7.42	59, 73, 100, 147, 158, 218	Isoleucine	7.39	73, 158, 100, 218, 75, 147, 74
5	Proline	7.47	59, 73, 142, 147	Proline	7.49	73, 142, 59, 75, 143, 66, 216
6	Glycine	7.55	59, 73, 86, 100, 133, 147, 174, 248	Glycine	7.57	73, 174, 86, 147, 59, 100, 133
7	Serine	8.05	73, 100, 147, 204, 218	Serine	8.10	73, 204, 100, 147, 75, 59, 218
8	Threonine	8.31	45, 57, 73, 101, 117, 129, 147, 219	Threonine	8.30	73, 117, 57, 219, 101, 147, 75
9	Methionine	9.45	61, 73, 100, 128, 147, 176	Methionine	9.48	73, 128, 176, 61, 147, 75, 100
10	Pyroglutamic acid	9.51	59, 73, 147, 156, 230, 258	Pyroglutamic acid	9.53	73, 156, 147, 84, 75, 258, 74
11	Cysteine	9.75	73, 100, 147, 218, 220	Cysteine	9.77	73, 100, 220, 147, 218, 132, 75
12	Glutamine	10.07	73, 128, 139, 147, 154, 155, 227	Glutamine	10.10	73, 154, 227, 147, 155, 139
13	Glutamine acid	10.24	73, 84, 128, 147, 156, 246	Glutamine acid	10.26	73, 128, 84, 75, 147, 156, 246
14	Phenylalanine	10.34	73, 100, 147, 192, 218	Phenylalanine	10.37	73, 192, 100, 147, 218, 75
15	Asparagine	10.66	73, 100, 116, 132, 141,188	Asparagine	10.70	73, 116, 132, 188, 147, 75, 141
16	Ornithine	11.73	73, 142, 147, 174, 100	Ornithine	11.66	73, 142, 174, 86, 59, 147, 100
17	Histidine	12.48	74, 100, 107, 154, 254	Histidine	12.55	73, 154, 254, 100, 74, 218
18	Tyrosine	12.57	73, 100, 147, 218	Tyrosine	12.52	73, 218, 100, 147, 75, 74
19	Cystathionine	14.26	73, 100, 128, 147, 218	Cystathionine	14.26	73, 128, 218, 174, 147
20	Tryptophan	14.36	73, 100, 147, 202, 218	Tryptophan	14.35	73, 202, 130, 74, 203, 100, 291
21	Oleamide	15.33	73, 75, 116, 128, 131, 144, 198	Oleamide	15.36	75, 131, 144, 116, 128, 198
22	Lactic acid	4.99	59, 75, 117, 147, 191	Lactic acid	4.95	73, 117, 147, 66, 75, 191, 148
23	Succinic acid	7.58	55, 73, 129, 147, 247	Succinic acid	7.60	73, 147, 75, 55, 247, 148, 129
24	Fumaric acid	7.83	75, 115, 143, 147, 245	Fumaric acid	7.89	73, 147, 245, 75, 143, 83, 115
25	Malic acid	9.19	55, 73, 75, 101, 133, 147, 233, 245	Malic acid	9.18	73, 147, 55, 75, 133, 233, 101
26	Glutaric acid	9.89	45, 55, 73, 112, 147, 156, 198, 170	Glutaric acid	9.92	73, 147, 198, 156, 89, 112, 229
27	Citric acid	11.76	45, 67, 73, 147, 273	Citric acid	11.79	73, 147, 273, 211, 73, 183
28	Glycerol	7.23	45, 73, 75, 103, 117, 133, 205	Glycerol	7.26	73, 147, 103, 117, 205, 218, 133
29	Glyceric acid	7.78	45, 73, 103, 117, 133, 189, 292	Glyceric acid	7.76	73, 147, 103, 117, 189, 133, 292
30	Fructose	12.18	73, 103, 117, 128, 133, 217, 307	Fructose	12.25	73, 103, 147, 217, 75, 133, 89
31	Glucose	12.36	129, 133, 147, 157, 160, 319	Glucose	12.34	73, 147, 103, 205, 160, 129, 319
32	Gluconic acid	13.06	73, 103, 117, 205, 217, 292, 333	Gluconic acid	13.11	73, 103, 217, 205, 292, 117, 333
33	myo-Inositiol	13.66	73, 103, 129, 147, 191, 217, 305	myo-Inositiol	13.65	73, 147, 217, 191, 129, 103, 305
34	Glucose 6-phosphate	15.01	73, 129, 147, 160, 299, 387	Glucose 6-phosphate	15.06	73, 147, 160, 299, 75, 129, 387
35	Adenosine-diphosphate	7.26	73, 133, 193, 207, 299, 300	Adenosine-diphosphate	7.25	73, 299, 133, 300, 193, 207, 211
36	Uridine	15.63	45, 73, 169, 217, 245	Uridine	15.44	73, 217, 169, 103, 224, 245, 258
37	Guanine	13.78	73, 74, 99, 100, 131, 352	Guanine	13.84	73, 147, 352, 99, 131, 264, 238
38	Adenosine	16.60	73, 103, 192, 217, 230, 236, 245	Adenosine	16.65	73, 103, 236, 230, 192, 217, 245

39	Benzoic acid	6.96	45, 51, 77, 105, 135, 179	Benzoic acid	7.01	105, 77, 179, 135, 51, 180, 136
40	Ethanolamine	7.15	59, 73, 86, 100, 133, 147, 174	Ethanolamine	7.17	73, 100, 86, 174, 147, 59, 133, 175
41	Urea	7.20	45, 73, 147, 171, 189	Urea	7.18	147, 189, 73, 148, 171, 74, 66, 99
42	Acetyl-glucosamine	13.58	73, 87, 117, 129, 173, 205	Acetyl-glucosamine	13.59	73, 129, 205, 75, 117, 157, 103

[a] Number of metabolites.; [b] Selected and tentatively identified primary metabolites based on variable importance in projection (VIP) value (>0.7) and *p*-value (<0.05) in both VIP1 and VIP2 by PLS-DA.; [c] Retention time.

Table S3. Comparison between beech mushrooms of three different sized samples (caps and stipes) and standard compounds equally analyzed by UHPLC-LTQ-IT-MS/MS

Beech mushroom sample							Standard compounds & Ref [3]					
No.[a]	Metabolites [b]	Ret (min) [c]	$[M–H]^-$	$[M+H]^+$	M.W.	MS^n $[M–H]^-$ fragment pattern (*m/z*)	Metabolites	Ret (min)	$[M–H]^-$	$[M+H]^+$	M.W.	MS^n $[M–H]^-$ fragment pattern (*m/z*)
44	Azelaic acid	8.36	187	–	188	187> 125> 97	Azelaic acid	8.38	187	189	188	187> 125> 97
45	Sebacic acid	9.40	201	–	202	201>183, 139	Sebacic acid	9.41	201	203	202	201> 183, 139> 111, 57
46	Hypsiziprenol A14	14.42	1214	1170	1169	1168>1098>1080, 1014	Hypsiziprenol A14 [d]	14.43	1214 [e]	1170	1169	1168> 1098> 1080, 1014
47	Hypsiziprenol A13	14.48	1082	1084	1083	1082>900>882, 816	Hypsiziprenol A13 [d]	14.49	1128 [e]	1084	1083	1082> 900> 928, 882, 816
48	Hypsiziprenol A12	14.56	996	998	997	996>814>956, 730	Hypsiziprenol A12 [d]	14.57	1042 [e]	998	997	996> 814> 796, 730

[a] Number of metabolites.; [b] Selected and tentatively identified primary metabolites based on variable importance in projection (VIP) value (>0.7) and p-value (<0.05) in both VIP1 and VIP2 by OPLS-DA.; [c] Retention time.; [d] mass spectra comparison with published paper ([3] Park et al., 2017).; [e] $[H+HCOOH–H]^-$

Supplementary Figure

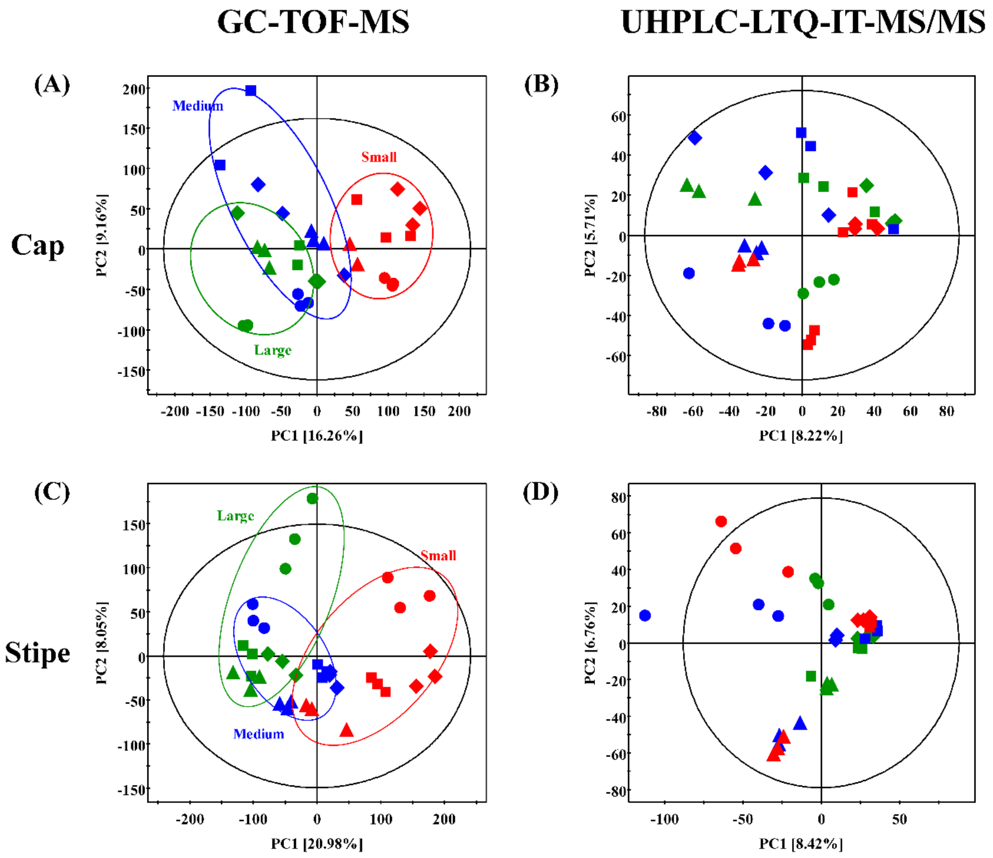

.

Fig. S1. Principal component analysis (PCA) score plots of caps (A and C) and stipes (B and D) collected according to different sizes of beech mushrooms analyzed by GC-TOF-MS (A and B) and UHPLC-MS/MS (C and D). Small size is indicated by red color, medium size is indicated by blue color, and large size is indicated by green color. ▲: KMCC03087, ●: KMCC03109, ■: KMCC03106, and ◆: KMCC03108

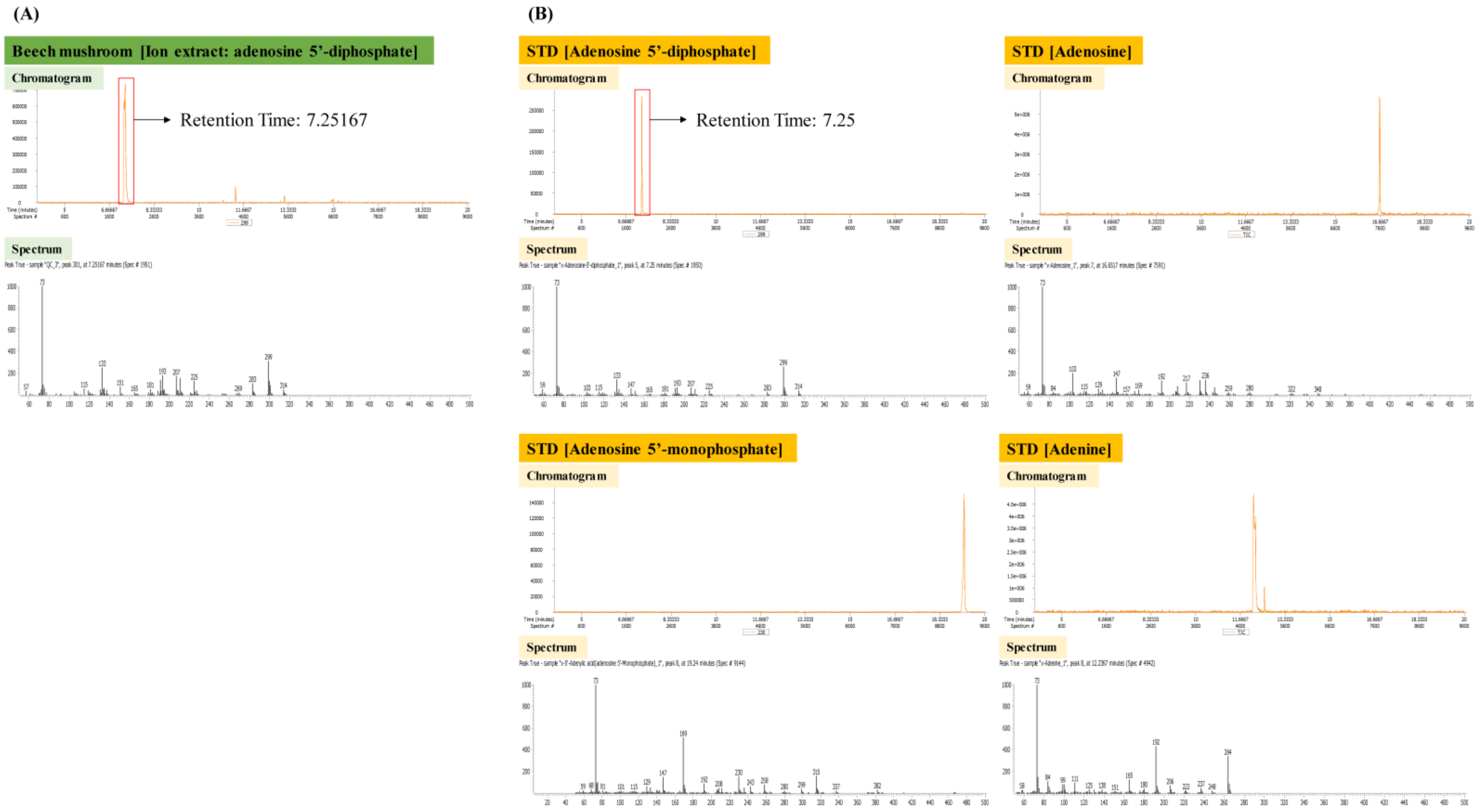

Fig. S2. The chromatogram and spectrum of adenosine 5′-diphosphate (ADP) from beech mushrooms analyzed by GC-TOF-MS (A). The chromatogram and spectrum of standard compounds (ADP, AMP, adenosine, and adenine) analyzed by GC-TOF-MS (B).
